# Melatonin-Hydrogel Delivery Systems for Effective Treatments of Periodontitis: Translational Perspectives from Material Designs to Clinical Applications

**DOI:** 10.3390/gels12090783

**Published:** 2026-09-01

**Authors:** Ting Liu, Li Zhang, Qiong Zhang, Qitao Zhao, Sisi Gou, Yifo Liu, Run Zhong, Guowei Mao, Hao-Ying Li

**Affiliations:** 1 Department of Stomatology, Guizhou Medical University, Guiyang 550004, China; 2 Institute of Pharmaceutical Science, King’s College London, London SE1 9NH, UK

**Keywords:** melatonin, periodontitis, hydrogels, periodontal tissue regeneration, antioxidant, anti-inflammatory, local drug delivery

## Abstract

Periodontitis is a chronic infectious disease characterized by progressive destruction of periodontal supporting tissues. Conventional treatments, including mechanical therapy and local drug delivery, are hindered by poor drug retention, burst release, and short duration of action. Melatonin (MT), an endogenous indoleamine, exhibits multi-target protective effects on periodontal tissues via reactive oxygen species scavenging, inflammatory regulation, bone metabolism balance, and microecological modulation; however, its clinical application is severely limited by a short half-life and rapid local clearance. To address these issues, hydrogels have been explored as carriers for periodontal drug delivery. These hydrophilic polymers offer biocompatibility, strong tissue adhesion, and sustained release properties, thereby enhancing local delivery and therapeutic outcomes. MT-hydrogel systems enable synergistic effects by integrating the modifiable functions of hydrogels with the pharmacological activities of MT. Their cooperative therapeutic mechanisms are critically categorized as antioxidant, anti-inflammatory, pro-regenerative, and microecological regulatory. This review summarizes key formulation determinants, including substrate selection, drug loading/sustained-release strategies, and functional modifications, and compiles recent data from in vitro, in vivo, and preliminary clinical studies. Furthermore, critical challenges in clinical translation are analyzed, and future research directions regarding formulation optimization and clinical application are proposed, providing a theoretical foundation for the development of effective MT-hydrogel systems for periodontitis therapy.

## 1. Introduction

Periodontitis is a chronic infectious disease initiated by dental plaque, characterized by progressive destruction of the periodontal supporting tissues. Its pathogenesis involves the dual effects of pathogenic bacterial invasion and abnormal host immune-inflammatory responses. Key pathological processes driving disease progression include excessive OS, sustained activation of inflammatory signaling pathways, bone metabolism imbalance, and impaired regenerative capacity of periodontal tissues [1,2,3]. Currently, conventional clinical treatment primarily focuses on the mechanical removal of dental plaque biofilms. However, for moderate-to-severe periodontitis, the mechanical therapy alone is insufficient to effectively modulate the host’s destructive inflammatory response, highlighting an urgent need for safe, effective, and targeted local adjunctive therapies [4,5].

MT (N-acetyl-5-methoxytryptamine) is an endogenous indoleamine widely distributed in the human body [6]. Early research has primarily focused on its role in pineal gland secretion, circadian rhythm regulation, and systemic metabolic modulation [7,8]. Recently, the synthesis and biological effects of MT in local tissues have garnered increasing attention. In particular, its expression and functional role in the oral microenvironment have become a research hotspot in periodontology [9]. Previous studies have confirmed that, in addition to systemic synthesis, MT can also be independently synthesized and secreted in local tissues such as salivary glands and oral mucosa, and is stably present in saliva and GCF, directly participating in the maintenance of oral microenvironmental homeostasis [10,11]. A negative correlation has been observed between the MT concentration in saliva and GCF and the clinical severity of periodontitis, suggesting that endogenous MT plays a positive role in maintaining periodontal homeostasis [12,13]. Exogenous MT exerts protective and regenerative effects on periodontal tissue through mechanisms consisting of scavenging ROS, regulating inflammatory pathways, modulating bone metabolism, and promoting the differentiation of periodontal stem cells [14,15,16]. However, the clinical use of MT is limited by its short half-life, rapid clearance from the local sites, and difficulty in maintaining sufficient drug concentrations onsite over an extended time period [17,18].

Hydrogels, as an advanced drug delivery platform, can overcome these limitations [19,20]. As 3D reticular hydrophilic polymers, hydrogels can adapt to the special anatomical structure of periodontal pockets, enabling local sustained drug release and targeted drug delivery. The adhesiveness of hydrogels mitigates the rapid loss of MT, and their capability for controlling drug release compensates for its short half-life [21,22]. The current drug delivery systems have unnegligible limitations for the treatment of periodontitis. Antibiotic hydrogels readily induce bacterial resistance and disrupt the normal oral microecology; growth factor hydrogels suffer from poor stability, high cost, and potential immunogenicity; And chlorhexidine formulations are prone to causing adverse effects such as tooth staining and taste alterations [23,24]. In recent years, the MT-hydrogels have garnered increasing attention at the intersection of oral biomaterials and periodontal disease therapy.

**Figure 1:**
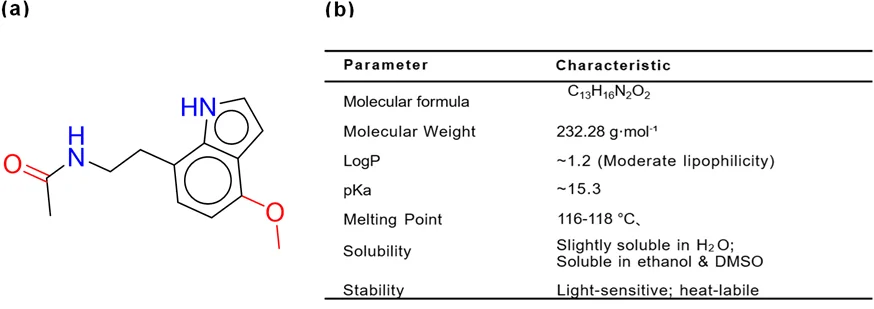
MT. (**a**) Chemical structure; (**b**) Key physicochemical properties.

The MT chemical structure (Figure 1a) and physicochemical properties (Figure 1b) have direct implications for formulation design. MT is characterized by a molecular formula of C_13_H_16_N_2_O_2_, a molecular weight of 232.28 g·mol^−1^, and a moderate lipophilicity (LogP ≈ 1.2). Its pKa value is approximately 15.3, corresponding to the indolic N-H proton, while the melting point falls within the range of 116–118 °C. In terms of solubility, MT is only slightly soluble in water but exhibits good solubility in organic solvents such as ethanol and dimethyl sulfoxide, which is a critical consideration for formulation design. Importantly, MT is both light-sensitive and heat-labile, necessitating strict protection from degradation during storage and administration [25,26]. These properties, combined with its short half-life and rapid local clearance [17,18], create a compelling case for a carrier system that can: (i) protect MT from environmental degradation; (ii) sustain drug release at the target site; and (iii) maintain therapeutic concentrations over extended periods. Hydrogels offer a versatile platform to meet these requirements.

This review follows a progressive framework from pharmacological basis and carrier design rationale to evidence stratification and translational considerations. We first outline the key pharmacological actions of MT on periodontal tissues, with particular attention to its concentration-dependent biphasic effects—a critical yet often overlooked constraint for sustained-release design. We then elaborate on the biological rationale for hydrogel carrier design tailored to the unique constraints of the periodontal pocket. Based on this foundation, we critically evaluate existing evidence through hierarchical categorization, distinguishing direct periodontal validation from extrapolated findings in non-periodontal models. By synthesizing common deficiencies in current studies, we identify priority directions for future research. This review aims to provide a conceptual reference for the rational design and clinical translation of MT-hydrogel delivery systems for effective periodontal therapy.

This review qualitatively synthesizes heterogeneous evidence rather than performing a meta-analysis. Within this scope, we searched databases, including PubMed/MEDLINE, Web of Science, Scopus, Embase, and CNKI, for peer-reviewed articles published up to July 2026. The search was performed using keyword combinations, including “melatonin”, “hydrogel”, “gel”, “scaffold”, “drug delivery”, “periodontitis”, “periodontal”, “alveolar bone”, and “periodontal ligament”. Reference lists were manually cross-referenced.

Given the narrative nature of this review, formal protocol registration and risk-of-bias assessments were omitted. We focused on prioritizing in vitro, in vivo, and clinical studies, and classified the evidence into four tiers: Category A (Direct Evidence): Investigations incorporating MT, hydrogel/scaffold carrier systems, and specific periodontal experimental or clinical models. Category B (Mechanistic Evidence): Periodontal studies assessing MT interventions in the absence of hydrogel vehicles. Category C (Matrix Evidence): Periodontal studies utilizing hydrogel/scaffold delivery systems without MT incorporation. Category D (Translational Reference): Studies evaluating MT hydrogel formulations in non-periodontal models. Category C and D data are included exclusively to inspire biomaterial design and are designated as hypothesis-generating-representing evidence with extrapolative limitations rather than definitive proof of therapeutic efficacy in periodontal treatment.

## 2. Mechanisms of MT-Hydrogel Synergy in Periodontitis Treatment

MT exerts direct protective effects on periodontal tissues by targeting the core pathological processes of periodontitis. Hydrogels address issues of rapid drug loss and low delivery efficiency. The mechanisms of action primarily involve antioxidation/mitochondrial protection, anti-inflammation/immunomodulation, periodontal tissue regeneration, and periodontal microecological regulation.

### 2.1. Antioxidant and Mitochondrial Protective Effects

**Figure 2:**
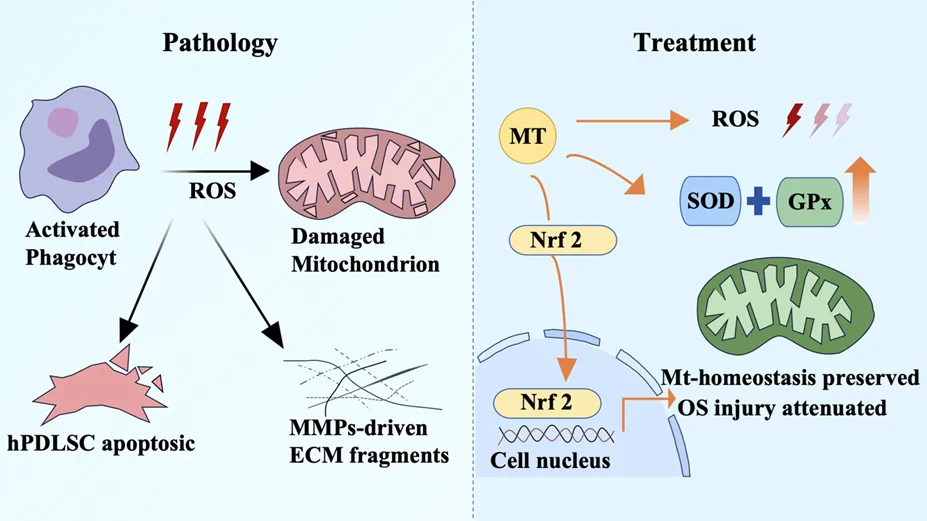
Antioxidant and mitochondrial-protective mechanisms of MT in the treatment of periodontitis. Schematic illustration of OS-mediated pathological events in periodontitis and the antioxidant and mitochondrial-protective mechanisms of MT. Black arrows represent pathological cascades driving periodontal tissue injury. Orange arrows denote pathways supported by Category B evidence. Activated phagocytes generate excessive ROS, which induces mitochondrial damage, hPDLSC apoptosis, and matrix-metalloproteinase-driven ECM fragmentation [27,28,29]. MT reduces ROS levels [32,33]. In ligature-induced experimental periodontitis in rats, MT treatment alleviated oxidative damage in periodontal tissues, accompanied by decreased MDA and elevated GSH levels [33]. MT also activates Nrf2 signaling to preserve mitochondrial homeostasis and counteract OS-associated periodontal damage [34,35].

OS is a core factor causing periodontal tissue damage [27]. In periodontitis lesions, ROS produced by activated phagocytes overwhelm local antioxidant capacity, leading to ECM degradation, mitochondrial DNA damage, and aggravated inflammatory responses [28,29]. Additionally, OS upregulates MMPs, accelerating alveolar bone resorption through inflammatory and apoptotic signaling pathways [30,31] (Figure 2).

MT exerts antioxidant and mitochondrial protective effects via multiple mechanisms. The indole ring scaffold of MT (Figure 1a) serves as the structural basis for its electron-donating capacity and free-radical scavenging activity. MT lowers ROS abundance [32,33] and ameliorates periodontal OS in experimental periodontitis, as evidenced by reduced MDA and increased GSH levels in periodontal tissues [33]. Furthermore, Xin et al. demonstrated that MT activates the Nrf2 signaling pathway to preserve mitochondrial homeostasis and attenuate OS-induced damage in periodontal tissues [34]. At the cellular level, it protects the mitochondrial structure and function of hPDLSCs, enhancing their survival rate and osteogenic potential under OS [35] (Figure 2). However, the antioxidant effects of MT alone in vivo can only last for hours, which cannot generate effective treatments for periodontitis. Hence, it is highly necessary to develop sustained drug release systems to achieve and maintain effective drug concentrations onsite for the continuous treatment of periodontitis up to several months as demanded.

### 2.2. Anti-Inflammatory and Immunomodulatory Effects

**Figure 3:**
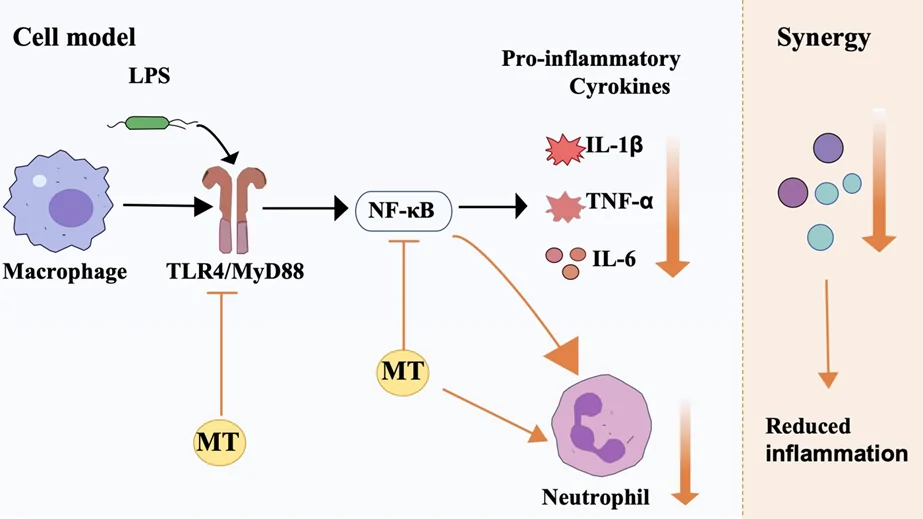
Anti-inflammatory and immunomodulatory mechanisms of MT in the treatment of periodontitis. Schematic overview of LPS-triggered TLR4/MyD88/NF-κB inflammatory cascades in macrophages and anti-inflammatory mechanisms of MT. Black arrows indicate pathological inflammatory processes of periodontitis. Orange arrows denote pathways supported by Category B evidence. LPS initiates TLR4/MyD88/NF-κB dependent signaling to activate NF-κB and promote proinflammatory cytokine production [33,36,38,42]. MT inhibits excessive TLR4/MyD88 signaling and NF-κB-related neutrophil-mediated inflammatory responses, contributing to the relief of periodontal inflammatory injury [37,39,40].

Abnormal chronic inflammation is a core pathological feature of periodontitis, characterized by elevated pro-inflammatory cytokine levels [36] and enhanced TLR4/MyD88 signaling activity [37], and triggers downstream NF-κB activation and further promotes inflammatory amplification in periodontal microenvironments [38]. MT protects periodontal tissues primarily by inhibiting overactivated TLR4/MyD88 signaling, reducing pro-inflammatory cytokine accumulation and alleviating alveolar bone resorption, thereby attenuating the development of periodontitis [37]. Beyond the TLR4/MyD88 cascade, MT also exerts anti-inflammatory effects by targeting the NF-κB-mediated NET signaling axis. MT has exhibited potent anti-inflammatory and immunomodulatory activities in preclinical periodontal models [39]. It inhibits NF-κB activation, reduces excessive NET formation, and suppresses subsequent neutrophil recruitment, ultimately mitigating periodontal inflammatory damage [40]. In vivo studies indicate that MT reduces neutrophil infiltration in periodontal tissues [41], decreases serum levels of pro-inflammatory cytokines [33,42], and attenuates periodontal inflammation and alveolar bone resorption (Figure 3).

However, MT also relies on sustained local drug concentrations to exert its anti-inflammatory properties. MT alone falls below therapeutic thresholds within hours following injection and cannot match the persistent inflammatory progression of periodontitis.

### 2.3. Bone Metabolism and Regeneration: Concentration-Dependent Effects

Periodontal tissue regeneration aims to restore the structural and functional integrity of alveolar bone, cementum, and periodontal ligament. In periodontitis, pathogen-derived LPS and inflammatory mediators disrupt the RANKL/OPG signaling axis, enhancing osteoclast activity while suppressing osteogenic differentiation, thereby compromising tissue regeneration [43,44,45].

A broader evidence base exists for MT alone in periodontal cells and animal models [46]. In vitro, MT induces osteogenic differentiation of human periodontal ligament stem cells and upregulates ALP, OCN, RUNX2 [15,47]; under inflammatory conditions, it preserves stem cell function through autophagy-related TMEM110 and YAP pathways [9]. In rat periodontitis models, MT suppresses osteoclastogenesis by lowering RANKL and raising OPG [48,49], and micro-CT analyses confirm reduced alveolar bone loss in treated animals [50].

Notably, MT’s osteogenic effects are concentration-dependent: physiological levels (≤1 nM) suppress osteogenic differentiation of hPDLSCs, accompanied by reduced intracellular ATP, increased ROS, and downregulation of MFN2 and TOM20, whereas pharmacological concentrations (1 μM) promote osteogenesis and upregulate osteogenic markers [51]. This biphasic behavior imposes explicit design constraints on hydrogel delivery systems: the initial burst release must not raise local concentrations into the suppressive range, nor should later release decay below physiological levels where osteogenesis may be impaired rather than promoted. An ideal release profile should maintain local concentrations within the pharmacological window—precisely the core objective of hydrogel carrier design.

**Figure 4:**
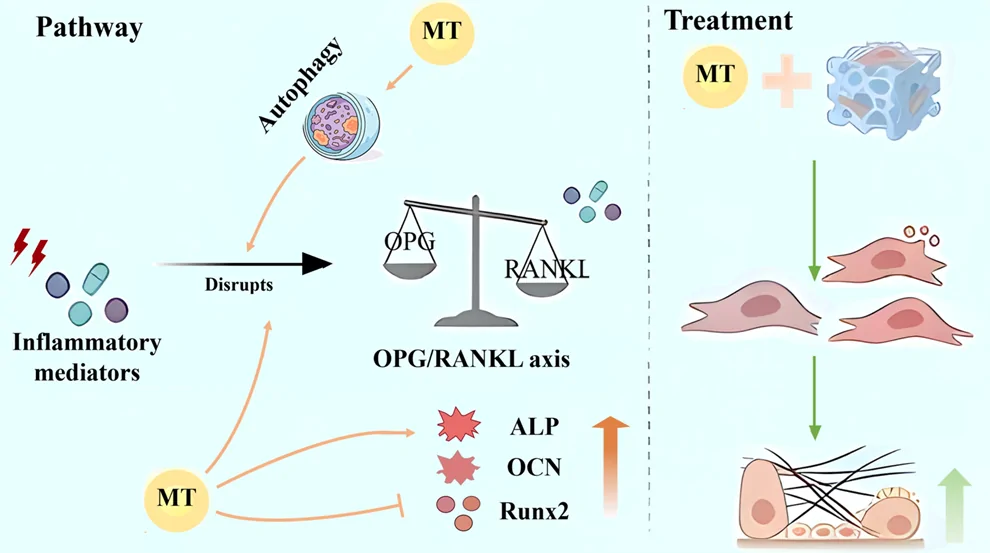
Synergistic action of MT-hydrogel on periodontal tissue regeneration. (**Left** **panel**): Proposed pathways. Pathological signaling is shown in black—LPS and inflammatory mediators disturb the RANKL/OPG axis, increasing osteoclast activity while reducing osteogenic differentiation [43,44,45]. Orange arrows denote Category B evidence. Free MT promotes osteogenic differentiation and upregulates ALP, OCN, and RUNX2 [15,47]; in rat periodontitis models, it suppresses osteoclastogenesis by lowering RANKL and increasing OPG [48,49], and reduces alveolar bone loss [50]. MT also preserves stem cell function under inflammatory conditions through autophagy-related TMEM110 and YAP signaling [9]. At physiological concentrations, MT suppresses osteogenic differentiation of hPDLSCs, accompanied by reduced intracellular ATP, increased ROS, and downregulation of MFN2 and TOM20 [51]. (**Right panel**): Category A evidence. TheMT@M2-exos/GelMA reduced alveolar bone loss in a rat periodontitis model [52]. A separate randomized trial using a simple 1% MT-gel with DFDBA, a non-engineered hydrogel, also reported the improved clinical outcomes [53]. An injectable MT-loaded scaffold promoted new bone formation in a canine furcation defect model [54]. Color code: Green, Category A; Orange, Category B; Black, pathological processes.

Remarkably, the two direct periodontal hydrogel studies have demonstrated that MT-loaded systems can indeed enhance alveolar bone regeneration in vivo, providing preliminary evidence that this concept is achievable: an MT-loaded GelMA hydrogel containing M2-exos (MT@M2-exos/GelMA) reduced alveolar bone loss in a rat periodontitis model [52]. A separate randomized trial using a simple 1% melatonin gel with DFDBA, not an engineered hydrogel, also reported improved clinical outcomes [53]. An injectable MT-loaded scaffold promoted new bone formation in a canine furcation defect model [54] (Figure 4).

### 2.4. Antimicrobial Activity and Microecological Modulation

**Figure 5:**
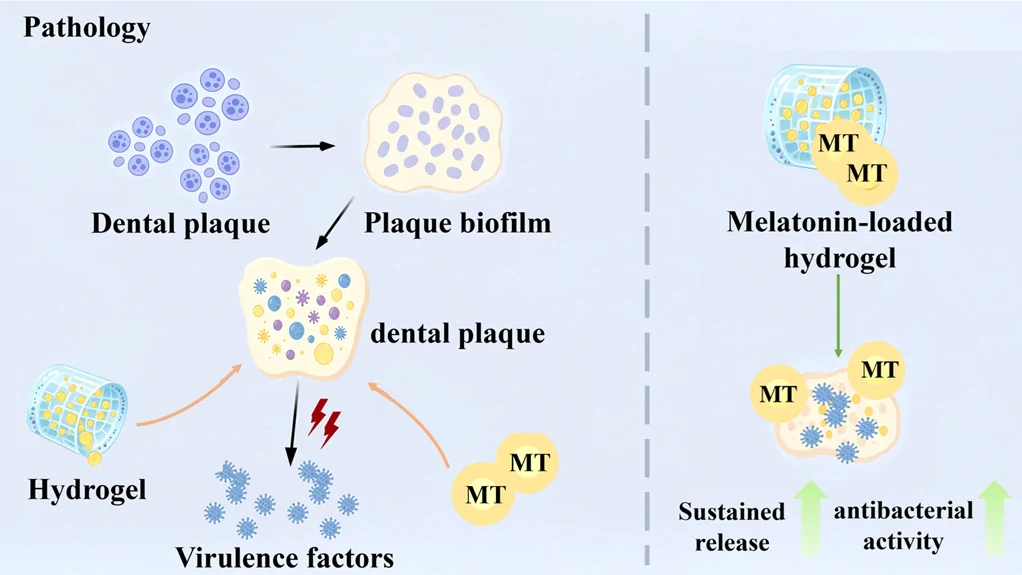
Periodontal microecology regulation by MT-hydrogel for periodontitis treatment. (**Left** **panel**): Pathological processes (in black). Bacteria adhere to the tooth surface and form plaque biofilm, which may subsequently mineralize into calculus [55,56]; these bacteria then release virulence factors, including gingipains and outer membrane vesicles, which directly damage periodontal tissues [55,56,57]. Orange indicates Category B/C evidence. MT alone exhibits antibacterial activity against periodontal pathogens [59]. The levels of salivary and gingival crevicular fluids negatively correlate to the severity of periodontitis [60], and the network pharmacology further suggests that MT may modulate MPO, MMP-8, and MMP-9 [61]. MT-free hydrogels have been shown to remain in the periodontal pocket and scavenge reactive oxygen species [62,63,64,65]. (**Right** **panel**): Category A evidence. A dual drug-loaded Gel-Alg/MINO/MT hydrogel inhibited biofilm formation, reduced inflammation, and promoted tissue reconstruction in an experimental periodontitis model [66]. Grey dashed lines denote unresolved issues, including the precise contribution of MT itself to biofilm control. Color code: Green, Category A; Orange, Category B/C; Black, pathological processes; Grey dashed, hypothetical.

Dental plaque biofilm and the overgrowth of key pathogens are the initiating factors of periodontitis. Bacteria such as *Porphyromonas gingivalis* and *Aggregatibacter actinomycetemcomitans* evade host immunity by forming biofilms and secrete virulence factors, including gingipains and outer membrane vesicles, that directly damage periodontal tissues and drive disease progression [55,56,57]. Regulating microecological homeostasis and inhibiting pathogenic biofilms are therefore critical components of periodontitis management [58] (Figure 5).

In vitro, MT exhibits antibacterial activity against prime periodontal pathogens [59]. Clinically, patients with periodontitis show reduced MT levels in saliva and gingival crevicular fluid, and these levels correlate negatively with disease severity, suggesting an association between endogenous MT and microecological homeostasis [60]. However, MT’s direct antibacterial activity is considerably weaker than that of conventional antibiotics.

This limitation provides the theoretical basis for the “MT + antibiotic” co-delivery strategy discussed in Section 3.3. The direct periodontal hydrogel study (Figure 5) has validated this concept by co-loading MT with MINO, achieving synergistic biofilm eradication and inflammation resolution [66]. The study tested a dual drug-loaded Gel-Alg/MINO/MT composite hydrogel in an experimental periodontitis model and in a three-dimensional, multispecies biofilm model. The hydrogel inhibited biofilm formation, reduced pro-inflammatory cytokines, and scavenged reactive oxygen species in vitro; in vivo, it relieved periodontal inflammation and promoted tissue reconstruction [66]. However, as the system also contained MINO, the specific contribution of MT remains to be isolated.

## 3. Material Designs and Performance Optimizations of MT-Loaded Hydrogels for Periodontitis Treatment

The material design and performance optimization of MT-loaded hydrogels are core to addressing the complex pathological microenvironment of periodontitis and improving local therapeutic efficacy. This section systematically elaborates on three aspects: (1) Matrix material selection that balances biocompatibility, mechanical stability, and tissue adhesion; (2) Drug loading strategies tailored to MT’s physicochemical properties and periodontal delivery needs; (3) Functional modifications that enhance targeting, regenerative capacity, and antibacterial activity. These designs collectively enable MT-hydrogel systems to achieve safe delivery, sustained release, and precise therapy for periodontitis.

### 3.1. Matrix Material Design of MT-Loaded Hydrogels

**Figure 6:**
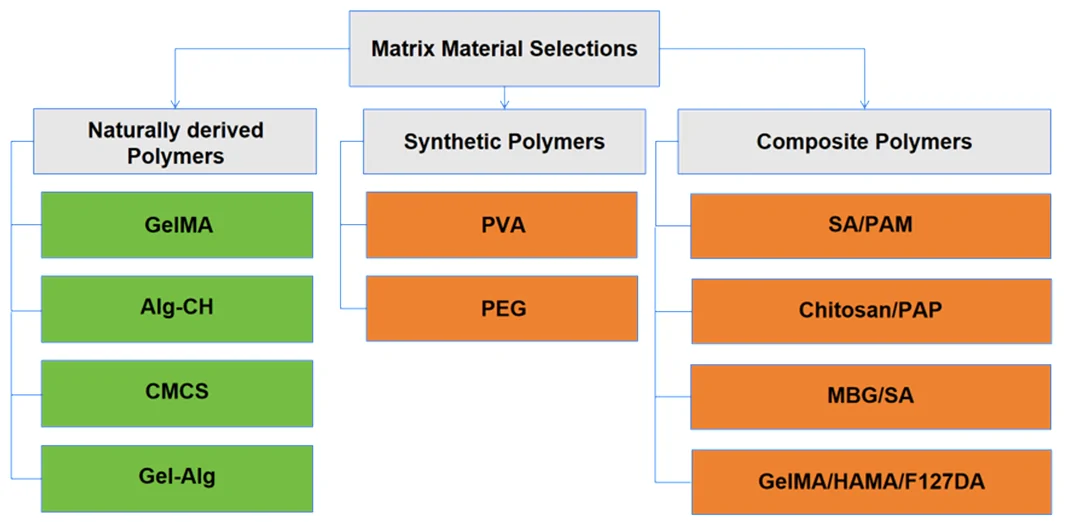
Selections of matrix materials for constructing MT-loaded hydrogel delivery systems. Three major matrix categories, including naturally derived polymers, synthetic polymers, and composite polymers, are summarized. Representative delivery systems consist of GelMA [52], Alg-CH [54], CMC [62], and Gel-Alg [66] for naturally-derived polymers, SA/PAM [68], chitosan/PAP [69], MBG/SA [70], and GelMA/HAMA/F127DA [71] for composite polymers, and PVA [72] and PEG [73] for synthetic polymers. Background color code for EC: Green, Category A; Orange, Category D.

The selection of hydrogel matrices is a fundamental prerequisite for determining the biocompatibility, drug loading capacity, sustained release behavior, and clinical applicability of drug delivery systems. Local drug administration for periodontitis requires the matrix to possess five characteristics: (i) Strong wet-tissue adhesion to resist salivary flushing; (ii) Injectability or in-situ gelation to conform to irregular pocket geometry; (iii) Controlled degradability matching the treatment duration; (iv) Mechanical resilience to withstand masticatory stress; and (v) Biosafety for long-term mucosal contact [67]. At present, matrices for MT loading are mainly divided into three categories: naturally derived polymers, synthetic polymers, and composite polymers (Figure 6). Figure 6 uses a color-coded scheme aligned with the evidence categories defined in the Introduction. In the following, we systematically summarize each category from the aspects of selection basis, structural characteristics, drug loading adaptability, and clinical applicability.

Naturally derived polymers offer wide availability, high biocompatibility, non-toxic degradation products, and tissue affinity [61]. Among the naturally derived polymer systems investigated as MT carriers, several have been evaluated directly in periodontal contexts.

GelMA (Figure 6) is a modified natural polymer derivative obtained by chemically modifying native gelatin with methacrylic anhydride. It retains the excellent biocompatibility and RGD cell-adhesion motifs of natural gelatin, while the introduction of photosensitive groups confers tunable photocrosslinkability, enabling in situ gelation within periodontal pockets upon ultraviolet or visible light irradiation to conform closely to irregular defect morphologies. Its three-dimensional network supports sustained release of MT@M2-exosomes, achieving a cumulative release of 85.12% over 48 h [52], thereby effectively prolonging the local retention of therapeutic agents at inflammatory sites and compensating for the short half-life of MT.

The Gel-Alg dual-network hydrogel combines gelatin, which provides cell-adhesion motifs and biocompatibility, with sodium alginate, which confers injectability and post-injection shape retention via ionic crosslinking, thereby enabling minimally invasive and precise filling of irregular periodontal pockets. The uniform porous network, with pore sizes of approximately 126 μm within the optimal range of 50–300 μm, balances drug diffusion and cellular infiltration. This system achieves an encapsulation efficiency of 84.9% for MT and a cumulative release of approximately 84.9% over 10 days, substantially higher than that of co-loaded MINO (61.6%), likely attributable to MT’s moderate lipophilicity and strong intermolecular affinity for the Gel-Alg network [66]. In a rat ligature-induced periodontitis model and a three-dimensional multispecies biofilm assay, the system inhibited biofilm formation by over 90%, reduced inflammatory cell infiltration, and promoted tissue reconstruction [66]. Notably, drug loading extended the degradation period from 6 to 14 days, suggesting that enhanced structural integrity supports sustained local delivery.

However, single-component naturally derived polymer systems are commonly constrained by inadequate mechanical strength, overly rapid degradation, and short release durations, necessitating composite modification or crosslinking enhancement to improve overall performance [74]. Synthetic polymer-based hydrogel composites possess tunable mechanical and degradable properties compared with naturally derived polymer hydrogels and can be engineered to realize sustained local drug delivery in periodontal defects [19]. However, direct evidence for synthetic polymer-based MT hydrogels in periodontal models remains absent.

Composite polymer matrices, which integrate naturally derived and synthetic components along with functional fillers, combine bioactivity, mechanical tunability, and synergistic multifunctionality (including antibacterial, anti-inflammatory, and osteogenic effects) [75]. The CAPA dual-network hydrogel, composed of naturally derived alginate and synthetic polyacrylamide, combines the biocompatibility and hemostatic properties of alginate with the mechanical resilience of polyacrylamide. Its interpenetrating network structure enabled a cumulative MT release of approximately 60% over 7 days, demonstrating enhanced structural stability and drug retention capacity [68]. The chitosan/PAP composite nanoparticles integrate a naturally derived polysaccharide matrix with a synthetic conductive triblock copolymer. Benefiting from a high porosity of 89.16% and a zeta potential of +52.9 mV, they achieved sustained drug release over 14 days with an initial burst of approximately 25%. Their metallic conductivity may further promote osteogenic differentiation through bioelectric signal transduction [69]. The MBG/SA composite hydrogel, incorporating MBG nanoparticles as fillers, increased the compressive modulus from approximately 0.5 MPa to 2.75 MPa, while the mesoporous structure served as a drug reservoir, enabling sustained MT release for up to 28 days [70].

In summary, the composite strategy achieves a triple synergy: naturally derived components provide bioactivity and biocompatibility, synthetic components confer mechanical strength and structural tunability, and functional fillers enable controlled release and synergistic therapeutic effects. This integration delivers comprehensive performance unattainable by single-component systems.

### 3.2. Strategies for Drug Loading and Sustained Release

**Figure 7:**
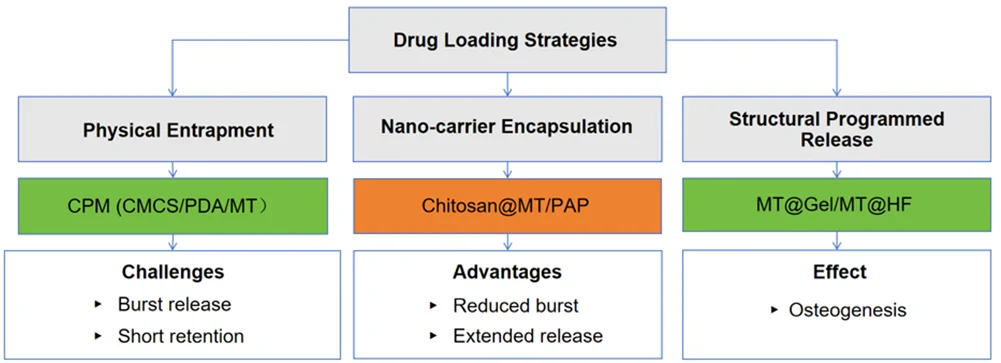
Drug-loading strategies for MT-hydrogel delivery systems. Three main strategies are presented: physical entrapment represented by CPM [62], nano-carrier encapsulation exemplified by chitosan@MT/PAP [69], and structural programmed release strategy signified by MT@Gel/MT@HAMA-F127DA composite hydrogel [71]. Background color code for EC: Green, Category A; Orange, Category D.

The design of MT loading strategies is jointly constrained by the inherent physicochemical properties of MT and the complex pathological microenvironment of periodontitis. Periodontal pockets feature high humidity, continuous salivary flushing, dynamic masticatory mechanical stimulation, acidic pH, and elevated ROS levels, which demand delivery systems with low burst release, long-term retention, inflammation-responsive drug release, and clinical operability. More importantly, MT’s own molecular characteristics determine the feasibility and performance of each loading approach. Based on the above requirements, the loading of MT into hydrogels to form drug delivery systems consists of the strategies of physical entrapment, nano-carrier encapsulation, and structural programmed release strategy, as shown in Figure 7.

Physical entrapment loads MT into the hydrogel network through non-covalent physical encapsulation, which is straightforward and avoids chemical modification, thereby preserving the pharmacological activity of the drug to the greatest extent. However, this strategy inherently suffers from pronounced burst release and short-term release kinetics, particularly in the acidic microenvironment of periodontitis, where the release rate of MT is extensively accelerated. The CPM hydrogel demonstrated pH-responsive MT release behavior in vitro, with cumulative release reaching 98% within 24 h at pH 5 (mimicking inflamed periodontal tissues) versus > 90% at pH 7 (healthy conditions) [62], which fails to meet the requirement of sustained release over several weeks for periodontal therapy.

Nano-encapsulation, which constructs nanoscale barriers (e.g., chitosan/PAP composite nanoparticles) around MT, effectively mitigates burst release and prolongs the release duration [69]. The chitosan@PAP nanoparticles (diameter 161.6 nm, zeta potential +52.9 mV, porosity 89.16%) achieved sustained drug release over 14 days with an initial burst of approximately 25%, and their electrical conductivity may further promote osteogenic differentiation through bioelectric signal transduction. The use of nano-carriers to encapsulate MT can offer further advantages. Firstly, the MT is pre-loaded into nano-carriers that then work as an extra diffusion barrier to slow down drug release, compensating for the high diffusivity due to the lipophilicity of drug molecules. Secondly, the nano-carrier shell shields MT from photodegradation and enzymatic hydrolysis in the oral cavity, which then extends the half-life of the drug in situ. Thirdly, nano-sized particles with mucoadhesive or active targeting surfaces can improve their adherence to the irregular inner wall of periodontal pockets and resist salivary flushing and masticatory displacement, which can then prolong the retention time of drug molecules at local sites, hence reinforcing the therapeutic effects.

Structured programmed release strategies achieve multi-stage therapeutic needs through heterogeneous material design. Inspired by the dual-faced heterogeneity of Janus materials, Huang et al. [71] constructed a core-shell structured hydrogel with a shell layer loaded with a high concentration of MT (100 mM) and a core layer loaded with a low concentration (5 mM). The GelMA shell degrades rapidly (>80% release within 7 days) to achieve early high-concentration antibacterial effects, while the MHF core provides sustained release (~60% over 30 days) to maintain long-term low-concentration osteogenic promotion. This programmed temporal release strategy offers important reference value for the staged treatment of periodontitis.

### 3.3. Functional Modification Design

**Figure 8:**
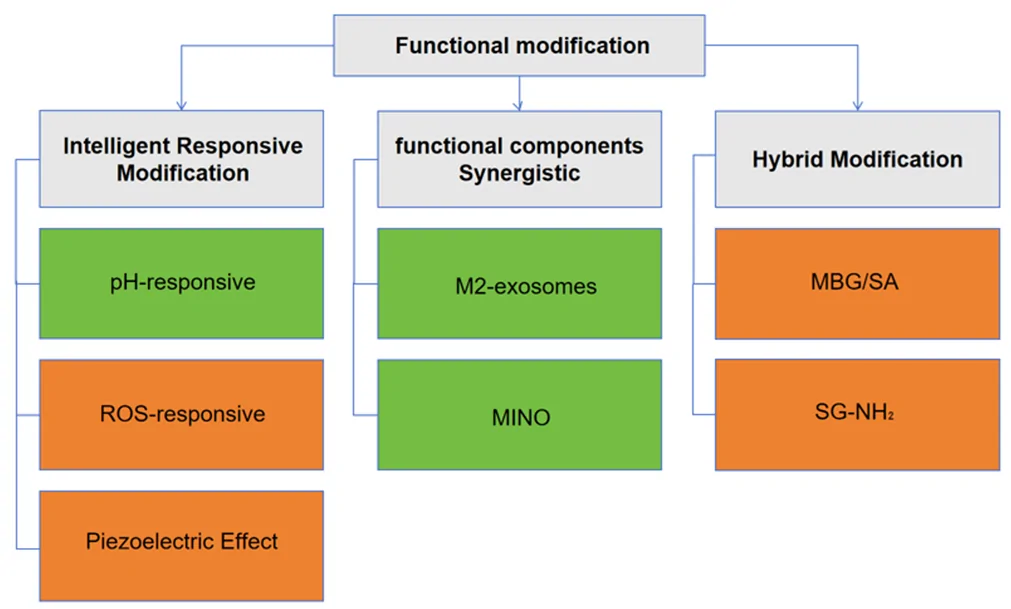
Functional modification strategies for MT-loaded hydrogel matrices. Three modification approaches include intelligent responsive modification, functional components synergistic modification, and hybrid modification. Representative examples consist of pH-responsive hydrogel [62], ROS-responsive hydrogel [72], and piezoelectric hydrogel [77] for intelligent responsive modification; M2-exosomes [52] and MINO [66] for functional components synergistic modification; MBG/SA [70] and 8-arm-PEG-SG/4-arm--PEG-NH_2_ [73] for hydrid modification. Background color code for EC: Green, Category A; Orange, Category C/D.

Unmodified matrices are difficult to meet the precise treatment needs in the complex microenvironment of periodontitis, so functional modification has become an important strategy to improve the targeting, responsiveness, adhesion, and therapeutic efficacy of MT-loaded hydrogels [76]. Modification strategies are carried out around the pathological characteristics of periodontitis, mainly including three categories: intelligent responsive modification, functional components synergistic modification, and inorganic-organic hybrid modification (Figure 8). The purpose is to enable hydrogels to sense the inflammatory microenvironment, enhance drug targeting, strengthen tissue regeneration, improve antibacterial capacity, and ultimately achieve more efficient and safer local therapeutic effects.

Intelligent responsive modification aims to enable hydrogels to achieve on-demand drug release in response to changes in the local periodontal microenvironment. Based on pH-responsive Schiff-base crosslinking constructed from the amino groups of carboxymethyl chitosan and the aldehyde groups of OD, the CPM hydrogel achieved a cumulative release of 98% within 24 h at acidic periodontal pocket pH 5, notably higher than that under neutral conditions (<90%, pH 7) [62], thereby enabling targeted delivery to inflammatory lesions.

Functional component synergistic modification introduces complementary components such as antibiotics and exosomes to compensate for the functional limitations of MT as a single agent. M2 exosomes synergize with MT to regulate macrophage polarization from M1 to M2 in a rat ligature-induced periodontitis model, downregulate the RANKL/OPG ratio, and considerably reduce the mRNA expression levels of IL-1 and TNF-α [52]. The gelatin-alginate hydrogel co-loaded with MINO and MT enhances MINO biofilm penetration by increasing bacterial membrane permeability through MT, while scavenging reactive oxygen species to attenuate MINO-induced cytotoxicity, achieving over 90% inhibition of multispecies periodontal biofilms and a DPPH radical scavenging rate of 92.2%. Furthermore, the complementary release kinetics, with zero-order sustained release for MINO and initial burst release for MT, further optimize the synergistic therapeutic outcome [66].

Functional modification designs derived from non-periodontal models (Category D evidence), although not yet validated in periodontal-specific models, offer valuable design principles that can inform the development of MT-loaded hydrogels for periodontitis. The ROS-responsive hydrogel crosslinked via phenylboronic ester bonds exemplifies an on-demand release design, achieving a cumulative MT release of 33–56% over 7 days under OS conditions (0.25–0.5 mM H_2_O_2_), representing an approximately 11.8-fold increase compared to the non-oxidative PBS control (4.72%) [72]. This design aligns with the high-ROS microenvironment characteristic of periodontitis, enabling lesion-specific drug delivery while minimizing systemic exposure. The piezoelectric hydrogel reinforced by ZnO/ZnS heterojunctions can convert mechanical stimuli from mastication into stable electrical signals (approximately 150 mV), thereby promoting osteogenic differentiation of periodontal ligament stem cells and inducing macrophage polarization toward the M2 phenotype [77]. This mechano-electrical coupling mechanism provides a design rationale for multifunctional delivery systems adapted to the dynamic oral microenvironment. Incorporation of MBG into SA hydrogels dramatically elevates the compressive modulus from approximately 0.5 MPa to 2.75 MPa at 90% strain, while the mesoporous structure serves as a drug reservoir enabling sustained MT release for up to 28 days [70]. This mechanical reinforcement approximates the load-bearing requirements of periodontal defects, where the hydrogel must withstand masticatory forces during the healing process. Meanwhile, the PEG@MT hydrogel, formed via amine-NHS crosslinking, achieves robust wet tissue adhesion with a fracture force of approximately 7 N and provides sustained MT release over 5 days [73]. This dual functionality of strong adhesion and sustained delivery is particularly critical for periodontal pockets, which are continuously subjected to salivary flow and mechanical disturbance.

The representative MT-loaded hydrogel systems discussed above are summarized in Table 1, in which their key formulation parameters are compared.

### 3.4. Rheological Properties of Hydrogels

The rheological properties of hydrogels directly influence clinical maneuverability and in vivo retention—both critical for effective MT delivery in the periodontal pocket [78]. Three rheological parameters are particularly relevant.

Shear-thinning behavior ensures injectability through fine-gauge needles into narrow periodontal pockets. Under syringe shear stress, the viscosity of gels decreases to produce a smooth flow for injection, while the gels become viscous again upon the removal of shear stress, which then allows the network rapidly recovered. The Alg-CH/β-TCP composite hydrogel (proven as an MT carrier in a canine periodontal model) demonstrates smooth, continuous injectability without interruption [54], while the PTD-MT hydrogel (tested in a non-periodontal myocardial model) exhibits similar shear-thinning behavior [72].

Gelation kinetics determine the time window for liquid-to-solid transition. Rapid gelation may cause needle clogging; slow gelation risks precursor washout by saliva. Thermo-responsive systems enabling rapid sol-gel transition at physiological temperature are particularly advantageous for adapting to irregular defect morphology [73].

**Figure 9:**
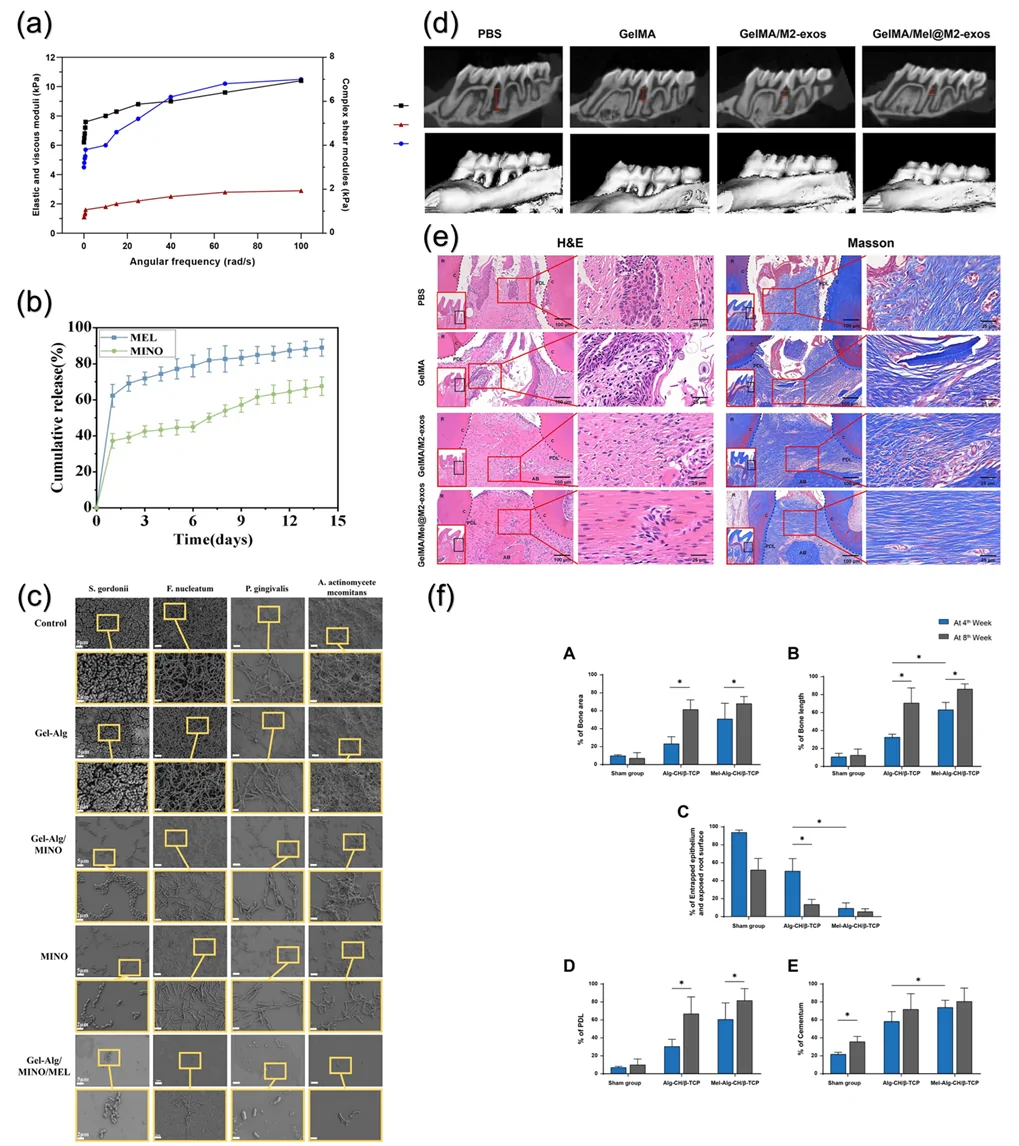
Representative experimental data from published Category A studies. (**a**) Rheological characterization of the Alg-CH/β-TCP composite hydrogel showing elastic modulus (G′) dominating viscous modulus (G′′), indicating readily injectable gel behavior. Reproduced from [54] with permission. (**b**) Cumulative release profiles of MINO and MT from the Gel-Alg/MINO/MT hydrogel over 10 days. Reproduced from [66] with permission. (**c**) SEM images of multispecies biofilms after treatment with Gel-Alg/MINO/MT, showing >90% inhibition. Reproduced from [66] with permission. (**d**) Micro-CT 3D reconstruction images of rat alveolar bone (red) in ligature-induced periodontitis models treated with PBS, GelMA, GelMA/M2-exos, and GelMA/MT@M2-exos. (**e**) H&E and Masson staining of periodontal tissue sections. AB = alveolar bone; C = cementum; PDL = periodontal ligament. Panels (d,e) are reproduced from [52] under a Creative Commons Attribution license. (**f**) Histomorphometric analysis of new bone formation in furcation defects at 4 and 8 weeks post-surgery, showing notably increased bone height in the MT-treated group at 4 weeks (* *p* < 0.05). Reproduced from [54] with permission.

Mechanical stability is reflected by the storage modulus (G′) relative to the loss modulus (G″) which determines resistance to salivary flushing, masticatory stress, and inflammatory exudate erosion. The Alg-CH/β-TCP composite hydrogel exhibits elastic-dominant behavior (G′ > G″) with no crossover over the tested frequency range, supporting shape integrity after injection [54] (Figure 9a). Similar elastic-dominant profiles are observed in MT-PTD and MT-PEG hydrogels, though these systems have been tested only in non-periodontal models [72,73]. Critically, most rheological data for MT-loaded hydrogels derive from non-periodontal models and have not been validated under oral-mimetic conditions specifically, artificial saliva at 37 °C, cyclic mechanical loading simulating mastication, and the shear environment of gingival crevicular fluid flow. Systematic rheological characterization under such periodontal-mimetic conditions is essential for optimizing injectability, defect-site adaptability, and long-term retention of MT-loaded hydrogels.

Building upon the systematic discussion of hydrogel rheological properties in the preceding sections, Table 2 further summarizes the key mechanical performance parameters of MT-loaded hydrogels, intended to provide data support for the analysis of mechanical adaptability across different matrix materials and drug loading.

## 4. MT-Hydrogels for Treatments of Periodontitis: In Vitro, In Vivo, and Clinical Studies

### 4.1. In Vitro Studies

Limited recent studies have investigated MT-loaded hydrogel systems for periodontal applications, with in vitro findings summarized below. The Gel-Alg hydrogel can well control the release of MINO and MT over 10 days, with about 60% *w*/*w* liberated from the system for MINO and 80% w/w for MT (Figure 9b). Besides, the CPM hydrogel exhibited concentration-dependent antibacterial effects against *E. coli* and *S. aureus and* promoted fibroblast migration (79% wound closure at 48 h vs. 49% for blank) [62]. However, this study used non-periodontal cell lines and bacterial strains, limiting direct translatability to periodontal pathogens and periodontal tissue cells. The Gel-Alg/MINO/MT demonstrated > 90% inhibition against multispecies biofilms of four major periodontal pathogens (i.e., *P. gingivalis, F. nucleatum, S. gordonii,* and *A. actinomycetemcomitans*), downregulated TNF-α and IL-1β in LPS-stimulated macrophages, and showed DPPH scavenging activity of 92.2% [66] (Table 3) (Figure 9c). Notably, MT mitigated MINO-induced cytotoxicity, elevating cell viability from ~60% to > 80% [66].

The current in vitro evidence suffers from several limitations. Most studies employ non-periodontal cell lines—L929 fibroblasts or RAW264.7 macrophages—rather than periodontal ligament cells or gingival fibroblasts, raising questions about translatability to periodontal tissues [62,66]. The Gel-Alg/MINO/MT system is a dual-drug formulation, meaning observed biological effects cannot be attributed solely to MT [66]. Additionally, most studies use hydrogel extracts rather than direct cell-hydrogel contact, failing to recapitulate the physical and mechanical interactions that occur in vivo [62,66]. The signaling pathways through which hydrogel-released MT exerts osteogenic and immunomodulatory effects remain poorly characterized.

Regarding concentration considerations, in vitro studies report MT ranging from 1 pM to 150 µM. MT exhibits a biphasic dose-response: physiological levels (1 pM–10 nM) may suppress osteogenic differentiation of hPDLSCs, whereas pharmacological concentrations (1–100 µM) promote osteogenesis, with 20–50 µM identified as the optimal window; concentrations of ≥100 µM show diminished efficacy and reduced cell viability [15,47,51]. Notably, the MT loading concentration in hydrogel systems (e.g., 5–10 mg/mL in the CPM hydrogel, equivalent to 21.5- 43 mM) is substantially higher—by approximately 860- to 2150-fold—than these pharmacologically effective concentrations [15,47,62]. This large discrepancy reflects the fundamental design principle of sustained-release hydrogels: a high drug payload is necessary to maintain therapeutic local concentrations over an extended period, rather than to expose cells directly to such high concentrations. However, in vitro applied concentrations do not equate to local tissue concentrations achieved by hydrogel release—a distinction often overlooked, as a systematic correlation between loading parameters and achieved local concentrations is lacking [51,62,66]. Moreover, the optimal concentration for periodontal regeneration in vivo likely differs substantially from in vitro findings due to protein binding, tissue diffusion, and rapid clearance in the periodontal pocket [6,13]. Consequently, the loading concentration of a hydrogel cannot be directly compared with, or substituted for, the effective concentration of free MT in conventional in vitro assays—a critical distinction for interpreting preclinical data and guiding future formulation design.

### 4.2. In Vivo Studies

Existing in vivo studies have shown that MT hydrogels can alleviate OS and inflammation, promote tissue healing, and effectively inhibit alveolar bone resorption, reduce inflammation in periodontal tissues, regulate bone metabolic balance, and create a local microenvironment conducive to bone regeneration [,^52^,^54^,^66^,^82^]. Three Category A publications complement one another in their use of animal models (Table 4): Abdelrasoul et al. [54] utilized a model comprising 36 Grade II root bifurcation defects in six dogs to evaluate the in situ osteogenic potential of regenerative materials in surgically created defects; Cui et al. [52] and Yang et al. [66] utilized ligature-induced periodontitis models in rats to simulate chronic inflammatory alveolar bone resorption; the former focused on the regulation of the inflammatory microenvironment, whilst the latter introduced a three-dimensional biofilm evaluation system involving four bacterial species. Together, these three studies cover a comprehensive evaluation spectrum ranging from surgical defects in large animals to infectious defects in small animals.

With regard to bone regeneration, Abdelrasoul et al. [54] demonstrated that, four weeks post-surgery, the height of new bone and the amount of new dentin in the MT-loaded group were superior to those in the control group; by eight weeks, the new bone had approached the morphology of normal compact bone (Figure 9f). Cui et al. [52] used micro-CT to confirm that the bone volume fraction (BV/TV) and trabecular bone thickness were extensively increased in the GelMA/MT@M2-exos group. Yang et al. [66] also found that in the Gel-Alg/MINO/MT group, the distance from the enamel-dentin junction to the alveolar crest was much reduced, and the BV/TV ratio was markedly improved (Figure 9d).

With regard to anti-inflammatory and immunomodulatory effects, in a rat periodontitis model, treatment with GelMA/MT@M2-exos considerably reduced the expression of pro-inflammatory cytokines such as IL-1β, IL-6, and TNF-α in periodontal tissues, induced the polarization of macrophages from the M1 to the M2 phenotype, and reduced the infiltration of neutrophils and lymphocytes; immunohistochemistry further confirmed increased expression of cementin and odonto-related proteins [52]. Yang et al. [66] also observed a reduction in inflammatory cell infiltration in Gel-Alg/MINO/MT system, as confirmed by both H&E and Masson’s staining (Figure 9e).

In terms of safety, none of the three studies reported any significant systemic toxicity or local adverse reactions. Yang et al. [66] confirmed that cell survival rates in the Gel-Alg/MINO/MT group were much higher than those in the MINO monotherapy group (*p* < 0.05), indicating that MT can mitigate the cytotoxicity of MINO; no abnormalities were observed in H&E staining of major organs or in serum biochemical parameters. Abdelrasoul et al. [54] observed no redness, swelling, ulceration, or systemic toxicity in their canine model; Cui et al. [52] also confirmed the good cellular compatibility of GelMA hydrogels with M2-exos.

However, the above conclusions are all based on preliminary experimental data and are subject to significant limitations. Firstly, the acute surgical defect model in dogs cannot simulate the clinical context of chronic infectious inflammation; furthermore, the rat ligation model differs from human periodontitis in terms of anatomical structure, immune response, and the rate of bone remodeling. Secondly, the sample size and observation period were limited: only six dogs were included, and only three rats per group were analyzed via micro-CT; the longest observation period was merely eight weeks, which is insufficient to assess long-term stability and safety. Thirdly, the Gel-Alg/MINO/MT formulation is a dual-drug combination of MT and MINO, and its additive effect cannot be definitively attributed to MT alone. Therefore, the above findings still require validation through animal experiments with larger sample sizes and standardized modeling to systematically assess their long-term safety and stability of efficacy under different pathological conditions.

### 4.3. Preliminary Clinical Research Evidence

At present, clinical research into MT-based topical delivery systems for periodontitis remains at a preliminary exploratory stage, and the existing evidence exhibits significant heterogeneity in terms of formulation types. Previous systematic reviews have summarized the clinical evidence regarding the use of MT as an adjunct to non-surgical periodontal treatment, confirming that MT, when used as an adjunct to routine SRP, can meaningfully improve probing depth, clinical attachment level, probing bleeding, and levels of inflammatory cytokines (IL-1β, IL-6, TNF-α), while only causing minor adverse reactions [90]. In a randomized controlled trial using a basic polymer hydrogel formulation, Srinath et al. applied MT (1%)-hydrogel topically within periodontal pockets as an adjunct to mechanical scaling; The data proved excellent safety associated with this delivery system and displayed considerable improvements in both the gingival index and periodontal probing depth in patients with stage II periodontitis [18]. However, this evidence was derived from a simple hydrogel formulation rather than an engineered complex hydrogel system. Furthermore, a small number of studies involving engineered nanocomposite hydrogels have indicated that the topical application of MT loaded into nanoparticle-modified hydrogels can further enhance anti-inflammatory and antioxidant activity and promote osteogenesis [91]. It should be noted that the formulation types involved in the aforementioned studies exhibit fundamental differences in rheological properties, release kinetics, and in vivo retention behavior. Consequently, the evidence regarding their therapeutic efficacy cannot be directly compared across different formulations. At present, clinical research into the use of MT-hydrogels for the treatment of periodontitis remains relatively limited, consisting mainly of preliminary exploratory studies with small sample sizes or involving simple hydrogels and engineered hydrogels (Table 5).

### 4.4. The Conversion Gap

Although preclinical studies in models of osteoporosis, periodontal bone defects, and soft-tissue trauma have yielded preliminary evidence regarding the osteogenic, anti-inflammatory and antioxidant properties of MT, the anatomical, physiological, and mechanical distinctiveness of periodontal tissues poses an obstacle to the extrapolation of this evidence. The following tissue-specific limitations necessitate that findings from non-periodontal studies be interpreted as hypothesis-generating rather than definitive evidence of efficacy.

#### 4.4.1. Salivary Flow and Renewal of Gingival Crevicular Fluid

The oral cavity constitutes a hydrodynamic environment with a high clearance rate. At rest, the salivary flow rate is approximately 0.25–0.35 mL/min; when stimulated, it can exceed 1.5 mL/min, creating a sustained flushing effect on topically applied formulations (such as gels) [101]. More importantly, the gingival crevice/periodontal pocket is continuously perfused by GCF, and the flow rate of GCF is greatly increased under inflammatory conditions [102]. This rapid fluid exchange substantially shortens the effective residence time of the drug molecules within the periodontal pocket [103], while this limitation is virtually non-existent in static tissue compartments, such as the subcutaneous implantation sites. Consequently, pharmacokinetic data derived from non-oral models may overestimate the local retention capacity of MT in periodontal tissues.

#### 4.4.2. Subgingival Biofilm Barrier

Periodontitis is, in essence, a biofilm-mediated disease. Subgingival biofilm forms a highly structured EPS matrix that functions both as a physical diffusion barrier and a biochemical shield [104]. In vitro studies and non-oral in vivo experiments typically assess the antimicrobial and anti-inflammatory activity of MT in free-floating bacterial culture systems, single-species models, or surgical sites devoid of bacterial colonization, where the resistance to mass transfer faced by the drug is minimal. By contrast, the EPS matrix of subgingival biofilms can significantly reduce the effective concentration of the drug at the tissue interface through mechanisms such as electrostatic adsorption, chelation, and enzymatic degradation, thereby protecting the niche of pathogenic bacteria [105]. Consequently, data on MT permeability and activity obtained from cell cultures or sterile bone defects cannot be extrapolated to the environment of mature, colonized subgingival biofilms.

#### 4.4.3. Geometric Characteristics and Accessibility Limitations of Periodontal Pockets

Periodontal pockets are narrow, elongated, and deep sac-like cavities, the depth of which varies drastically between healthy conditions and severe periodontitis. This geometric morphology imposes severe limitations on the distribution and retention of topical formulations [106,107]. MT-Hydrogels injected into deep pockets with irregular shapes may fail to reach the pocket floor due to inappropriate rheological properties or insufficient injection pressure, and they may accumulate at the gingival margin or be displaced by increased GCF flow velocity, hence resulting in irregular tissue contours and exacerbated local inflammation [108]. Furthermore, furcation lesions and intraosseous defects exhibit complex 3D structures, which further worsen the geometric challenges associated with drug delivery to the periodontal cavities.

#### 4.4.4. Stresses in Masticatory Mechanics

Periodontal tissues are continuously subjected to functional loads. Masticatory forces generate intermittent pressure on the occlusal surfaces, which is transmitted via the periodontal ligament to the alveolar bone, inducing cyclic micro-movements [109]. Unlike static orthopedic implants or skin wound sites, periodontal tissues are continuously exposed to dynamic mechanical stresses during mastication, swallowing, and speech. This continuous biomechanical activity can displace or compress locally applied hydrogels, accelerating their mechanical fatigue, compromising the integrity of mucosal adhesion, and eventually causing the premature clearance of formulations, henceforward the loss of therapeutic efficacy [110,111].

#### 4.4.5. Enzymatic Degradation in the Oral Cavity

The oral cavity is highly enzymatic. Saliva contains amylase, lysozyme, peroxidase, and various proteases, while subgingival plaque is rich in bacteriogenic collagenase, gelatinase, and hyaluronidase [112]. These enzymes can degrade hydrogel carriers (e.g., gelatin, alginate, chitosan, etc.) and active pharmaceutical ingredients. Although MT is relatively stable, the structural integrity of the carrier is compromised by the combined action of oral peroxidase and ROS generated by polymorphonuclear leukocytes within inflamed periodontal pockets, thereby affecting the drug release kinetics [113]. Consequently, degradation rates and drug stability data obtained in a subcutaneous environment cannot predict actual performance within the oral cavity.

#### 4.4.6. Confounding Effects of Clinical Debridement

A key but often overlooked confounding factor is subgingival SRP, which is almost universally combined with topical medication in clinical periodontal trials [114]. Mechanical debridement, by disrupting the biofilm and removing calculus, can improve clinical attachment levels, probing depths, and inflammatory markers. In non-periodontal studies (such as those on MT treatment for osteoporosis or long bone defects), such background interventions are absent, allowing the drug’s effects to be isolated independently. However, in periodontitis trials, MT preparations have consistently been evaluated as an adjunct to SRP (Table 4); it is difficult, from both a statistical and clinical perspective, to separate the incremental pharmacological benefits of the drug from the mechanical effects of SRP. This implies that even if periodontitis trials yield positive outcomes, the therapeutic effect cannot be attributed solely to MT itself.

#### 4.4.7. The Epistemological Status of Non-Periodontal Evidence

As mentioned above, there are a number of tissue-specific variables, which consist of hydrodynamic clearance, the biofilm barrier, periodontal pocket geometry, masticatory stress, enzymatic instability, and mechanical debridement. These variables and their combinations can then form a robust translational barrier to render periodontal tissues in a pharmacologically unfavorable and mechanically unique environment. Consequently, data generated from non-periodontal models are valuable for formulating mechanistic hypotheses while insufficient to constitute conclusive evidence of MT’s therapeutic efficacy in human periodontitis. Future studies should employ preclinical models, especially related to periodontitis, and develop multi-center randomized clinical trials to generate sufficient reliable data to bridge the translational gap.

## 5. Conclusions and Future Perspectives

Despite its protective and reparative actions on the periodontium, MT is constrained by a short half-life and a propensity for local loss in clinic trials. Hydrogels, as an ideal local drug delivery carrier, overcome the formulation defects of MT. The MT-hydrogel composite systems can maintain an effective local concentration of MT, prolong the therapeutic time, provide a favorable microenvironment for periodontal tissue regeneration, and enhance the anti-inflammatory, antioxidant, and microecological regulatory effects of MT on tissue regeneration. In vitro, animal, and preliminary clinical studies have provided preliminary support that MT hydrogels can effectively inhibit alveolar bone loss, alleviate periodontal inflammation, promote periodontal tissue regeneration, and regulate periodontal microecology, with favorable biocompatibility in the tested models and safety, emerging as a promising host-modulated local therapy for periodontitis.

Despite the promising therapeutic potential, the clinical translation of MT-hydrogels still faces several critical challenges: Unstandardized material design and preparation protocols. The current MT hydrogel substrates, drug loading strategies, and functional modification methods are diverse, with no unified performance evaluation standards and preparation protocols, leading to inconsistent experimental results; and unclear optimal dosing regimen. The optimal concentration, administration frequency, and intervention cycle of MT-hydrogels for different severities and types of periodontitis are still unclear [115]; lack of long-term efficacy and safety data; insufficient combination with conventional periodontal therapy. The synergistic therapeutic effects with conventional periodontal therapy are still not fully explored.

Beyond the challenges associated with clinical translation, MT-hydrogels also possess inherent limitations that need to be further addressed. In pharmacokinetic considerations of MT, the risk of drug interactions arising from systemic absorption should not be overlooked. MT is primarily metabolized by CYP450 1A2, with possible contributions from CYP450 2C19 and 2C9 [116]; consequently, co-administration with potent inhibitors of CYP450 1A2, such as certain fluoroquinolone antibiotics, may significantly elevate plasma MT concentrations, and this type of interaction risk warrants due attention in clinical scenarios involving concomitant use of MT-hydrogels with other medications. Kanikkannan et al. systematically evaluated the skin sensitization potential of MT at concentrations of 0.5%, 2.5%, 5.0%, and 10.0% (*w*/*v*). The results showed that the stimulation index values in all treatment groups were close to 1, indicating that MT did not induce skin sensitization within the tested concentration range [117]. Furthermore, assessments by the European Chemicals Agency have similarly concluded that MT is not a skin sensitizer. The safety of drug-loaded hydrogels is highly formulation-dependent. In most reported studies, no adverse reactions or systemic toxicity were observed under the stringent selection of appropriate formulations [118,119], suggesting that hydrogel formulations should be carefully chosen according to specific application contexts to avoid local irritation. Studies have shown that the degradation rate of hydrogel matrices can be optimized through compositional modulation [118]; however, systematic investigations regarding the specific composition of degradation products and their potential biological effects remain scarce, constituting a gap in the current knowledge base. Existing preclinical evidence generally supports a favorable short-term safety profile for MT hydrogels; for example, in a study using MT-loaded tamarind chitin nanofiber hydrogels, MT remained chemically stable for at least 31 days [120]. Nevertheless, long-term data on the use of MT-hydrogels are considerably limited, and the variability in formulation composition as well as the potential for systemic absorption must be taken into account, underscoring the necessity of establishing long-term in vivo evaluation systems. In the field of wound healing, circadian rhythm disruption has been recognized as a critical factor affecting wound repair. Wu et al. developed an alginate-based dual-network hydrogel as an MT delivery system, and their findings demonstrated that this hydrogel promoted wound healing by modulating circadian rhythms, alleviating OS, and suppressing inflammatory responses, suggesting the application potential of MT-hydrogels in promoting wound healing through circadian rhythm regulation [68]. However, further investigation is required. Meanwhile, the biological effects of MT-hydrogels frequently exhibit a clear dose-dependent pattern, which can be manifested across multiple dimensions, including tissue regeneration promotion, anti-tumor activity, and anti-inflammatory responses. For instance, the MT-hydrogels system designed by Huang et al. demonstrated that a high dose of MT exerted an inhibitory effect on tumor cell growth, whereas a low dose promoted osteogenic differentiation [71]. This necessitates that the design of hydrogel delivery systems takes full account of the differential concentration requirements of MT dictated by the target indications.

**Figure 10:**
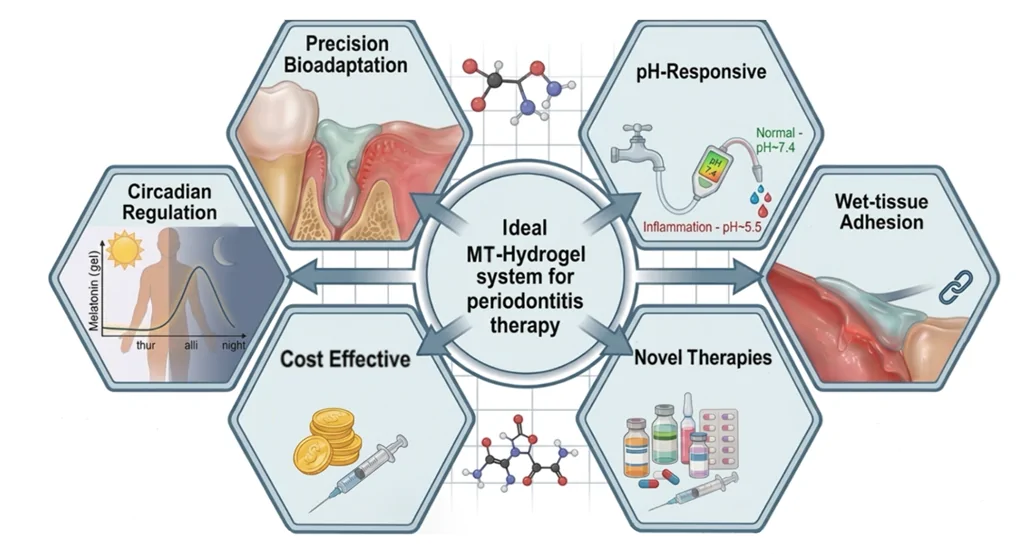
Desirable characteristics of ideal MT‑loaded hydrogel systems for periodontitis therapy. Six hallmark features are summarized, consisting of precision bioadaptation, pH‑responsive release, wet‑tissue adhesion, novel therapies, cost‑effective production, and circadian‑rhythm regulation [2,18,62,68,82,91,121,122,123]. These properties support microenvironment adaptation, chronobiological modulation and clinical feasibility.

To accelerate the clinical translation of MT-hydrogels and improve their therapeutic efficacy and clinical applicability, future studies should primarily encompass the following directions, as shown in Figure 10. (1) The morphological adaptation to irregular periodontal pocket anatomy is achieved through injectability and in-situ gelation; (2) the pH-responsive mechanism is designed based on the significant acidification characteristics of the periodontal disease microenvironment, which shifts from healthy states (pH 6.5–7.5) to inflammatory states (pH 5.0–6.5), thereby achieving inflammation-targeted intelligent drug delivery and self-adaptive release rate modulation [121]; (3) the wet-tissue adhesion property provides a stable carrier platform for localized and sustained MT release, thereby preventing drug loss due to saliva flushing [62]; (4) novel therapies such as those combined with the conventional periodontal therapy are explored, hence developing comprehensive treatment strategies for periodontitis [2]. A patent discloses a biocompatible paste formulated with MT, HA, tetracycline, and metronidazole for local management of chronic marginal periodontitis, in which the active components—MT, antibiotics, and an HA-based carrier—demonstrate synergistic interactions in biological environments, highlighting substantial promise for combining MT with traditional periodontal treatment modalities [122].The synergistic effects can be employed to develop personalized and multifunctional MT-hydrogels, to design MT-hydrogels tailored to the pathological characteristics of different types of periodontitis, and to construct multifunctional scaffolds capable of releasing growth factors and MT molecules at bone defect sites [82,123]; (5) optimizing clinical operability and reducing production cost, carrying out large-sample, multi-center clinical trials [18,91], screening low-cost naturally derived polymer substrates, and simplifying preparation processes to reduce large-scale production costs; and (6) deepening the research on circadian rhythm regulation, further exploring the mechanism of MT-hydrogels in regulating oral local circadian rhythm, and clarifying the role of circadian rhythm regulation in periodontitis treatment [68].

## Figures and Tables

**Table 1:** MT-Loaded Hydrogels: Key Design Parameters and EC.

Hydrogel System	Cumulative Dug Release (%, *w*/*w*), [Time]	Drug Release Conditions	Local Concentration/Dose	Application Site	Ref.	EC
MT@M2-exos/GelMA	85.12, [48 h]	PBS (pH 7.4), 37 °C	200 µL hydrogel per rat (containing MT@M2-exos)	Periodontitis	[52]	A
Alg-CH/β-TCP@MT	N/A	PBS (pH 7.4), 37 °C	3 mg/mL MT in hydrogel	Class II furcation defects (periodontitis)	[54]	A
CPM	>90, [24 h] (pH 5–7)	PBS (pH 5–7), 37 °C	5 or 10 mg/mL MT in hydrogel	Periodontitis	[62]	A
Gel-Alg/MINO/MT	~84.9, [10 d]	PBS(pH 7.4), 37 °C	40 mg/mL MT	Periodontitis	[66]	A
MT@CAPA	~60, [7 d]	PBS (pH 7.4), 37 °C	5 mM MT in hydrogel	Skin wound	[68]	D
Chitosan@MT/PAP	~75, [14 d]	PBS (pH 7.4), 37 °C	50 μMMT in hydrogel	Osteoporosis	[69]	D
MT-MBG/SA	~80, [20 d]	PBS (pH 7.4), 37 °C	50 µM MT in hydrogel; 2 µL/disc	Intervertebral disc regeneration	[70]	D
MT@Gel/MT@MHF (shell)	>80, [7 d]	PBS (pH 7.4), 37 °C	100 mM MT in shell hydrogel	Postoperative bone tumor reconstruction	[71]	D
MT@Gel/MT@MHF (core)	~60, [30 d]	PBS (pH 7.4), 37 °C	5 mM MT in core hydrogel			
MT-PTD-	33~56, [7 d]	PBS ± H_2_O_2_ (0.25/0.5 mM), 37 °C	150 µM MT; 30 µL/kg	Myocardial I/R injury	[72]	D
PEG@MT	~50, [2 d]; ~90, [5 d]	PBS (pH 7.4), 37 °C	~8.1 mg/mL	Liposarcoma resection wound	[73]	D

Abbreviations: d, days; h, hours;

**Table 2:** Mechanical Properties of MT-Loaded Hydrogels.

Drug-Hydrogels	Compressive Modulus	Tensile Strength	Swelling Ratio	Adhesion Strength	Refs.	EC
MT-CPM	N/A	N/A	Peaked at 30 min	Strong	[62]	A
Gel-Alg/MINO/MT	N/A	N/A	Decreased compared to Gel-Alg	Strong; Resistant to twisting, bending, and water	[66]	A
MT-CAPA		N/A	400%	N/A	[68]	D
MT-MBG/SA	0.5–2.75 MPa	Up to 0.5 MPa	Reduced as the increase in MBG in gels	N/A	[70]	D
MT-Gel/MT-MHF	0.1–0.75 MPa	MHF > Gel	~400%	N/A	[71]	D
MT-PEG	G′ > G″	N/A	N/A	Fracture force: 7 N	[73]	D

Nomenclature: G′, storage modulus; G″, viscous modulus.

**Table 3:** Summary of Periodontal-Related In Vitro Studies on MT.

Year	MT	Cell Line(s)	In Vitro Stimuli	Key Findings	Refs.	EC
2026	MT (11.32 mg/mL)	RAW264.7	LPS	Reduced MINO cytotoxicity, inhibited biofilm, and exerted anti-inflammatory and antioxidant effects	[66]	A
2025	MT (0–150 μM)	PDLSC	N/A	Promoted osteogenesis by modulating collagen deposition and calcium mineralization	[15]	B
2025	MT (0.5–5.0 µM)	Inflamed PDLSCs	LPS	Concentration-dependent osteogenesis; via autophagy/TMEM110	[9]	B
2024	MT (1–100 μM)	PDLSC	H_2_O_2_	Attenuated OS-induced senescence and promoted osteogenic differentiation via Hippo-YAP signaling	[79]	B
2023	MT (1–100 µM)	hPDLC	Osteogenic medium	Most effective at 1 µM; regulated mitochondrial function via TOM20	[35]	B
2023	MT@M2-exos	hPDLC, RAW264.7	LPS	Enhanced osteogenesis, induced M1-to-M2 macrophage polarization, and reduced ER stress-mediated apoptosis	[52]	B
2021	MT (1 μM)	PDLSC	N/A	Restored autophagy and attenuated long-term expansion-induced senescence via PI3K/AKT/mTOR	[80]	B
2020	MT (0–1 μM)	PDLSC	N/A	Restored/upregulated OPN and OCN levels and promoted osteogenic differentiation at pharmacological concentrations	[51]	B
2023	MT (5–10 mg/mL)	L929	N/A	Enhanced cell migration, antibacterial activity, and cytocompatibility	[62]	D
2020	MT (5–20%)	MC3T3-E1	H_2_O_2_	Inhibited mitochondrial OS via the SIRT3/SOD2 signaling pathway	[81]	D

**Table 4:** Summary of Periodontal-Related In Vivo Studies on MT.

Year	APIs	Delivery Systems	AnimalModels	Key Findings	Refs.	EC
2026	MINO, MT	Gel-Alg	Periodontitis (Rats)	Periodontal inflammation and promotion of tissue reconstruction	[66]	A
2023	MT	M2-exos/GelMA	Periodontitis (Rats)	Inhibited apoptosis and promoted bone formation	[52]	A
2022	MT	SA- Chitosan/β-TCP	Type II bifurcation defects (Dogs)	Inhibited epithelial ingrowth, accelerated formation of new bone	[54]	A
2025	MT	Injection	Periodontitis (Rats)	Periodontal regeneration	[9]	B
2024	MT	Injection	Periodontitis (Rats)	An alternative to amifostine	[83]	B
2024	MT	Photobiomodulation + Injection	Periodontitis (Rats)	Superior combination therapy than monotherapy	[84]	B
2025	Doxycycline	Nanocomposite thermosensitive hydrogel	Periodontitis (Rats)	Antibacterial, anti-inflammatory, promoted bone and periodontal regeneration, and targeted delivery	[85]	C
2024	Embelin	CMC-OD	Periodontitis (Rats)	Restored osteogenic function and alleviated bone loss	[86]	C
2025	MT	ROS-responsive injectable hydrogel	Myocardial I/R injury (Rats)	Reduced size of the infarct and promoted recovery of cardiac function)	[72]	D
2024	MT	Zn-Ga/mHA	Skull defects (Rabbits)	Strong antibacterial and bone-forming effects	[87]	D
2024	MT, Menstrual blood stem cells	Gelatin nanofibers	Cartilage defect(Rats)	Greater healing effect compared to single-polymer hydrogels	[88]	D
2023	MT	Hydrogel	Atrial fibrillation tissue box-shaped defect (Rats)	Good biocompatibility and self-healing	[89]	D

**Table 5:** Clinical Studies of MT-Based Formulations in Periodontitis.

Year	APIs	Delivery Systems	Disease(s)/Patient Number	Therapeutic Effects	Refs.	EC
2025	MT (1% *w*/*v*)	Gel	Periodontitis/24	Antioxidant potential	[18]	B
2025	MT (0.1% *w*/*v*)	Chitosan nanoparticles	Periodontal intraosseous defects/67	Repair of periodontal bone defects	[91]	B
2025	MT (1% *w*/*v*)	Gels/demineralized bone allograft	Bone defects/20	Treatment of periodontal defects	[53]	B
2024	MT (5% *w*/*v*),Vitamin C	Gel	Chronic periodontitis/100	Relieved periodontal inflammation	[92]	B
2024	MT (1% *w*/*v*)	Gel	Periodontitis/100	Lessened bone defects; Alleviated inflammatory responses	[93]	B
2023	MT (6 mg/day)	Oral tablets	Periodontitis & diabetes/55	Improved clinical outcomes as SRP adjunct	[13]	B
2023	MT (1% *w*/*v*), PRF	Gel/platelet-rich fibrin	Bilateral Grade II bifurcation defects/23	Repair of periodontal bone defects	[94]	B
2022	MT (1% *w*/*v*)	Gel	Bilateral intraosseous defects/22	Repair of periodontal bone defects	[95]	B
2017	MT (1% *w*/*v*)	Orabase cream	Periodontal disease/90	Improvement in gingival index and periodontal pocket depth	[96]	B
2026	N/A	Oscillating chitosan brush	periodontitis/38	Improved outcomes of subgingival implantation	[97]	C
2023	N/A	Arginine-glycine-aspartic acid composite hydrogel	bone defects/45	Improved outcomes of periodontal treatment	[98]	C
2015	N/A	Gel/rhFGF-2	bone defects/30	Healing of periodontal wounds	[99]	C
2025	MT (1% *w*/*v*)	Hydrogel	Foot ulcers/30	Treatment of ulcers	[100]	D

## Data Availability

No new data were created or analyzed in this study. Data sharing is not applicable to this article.

## Abbreviations

The following abbreviations are used in this manuscript:

3D	Three-dimensional
4-arm-PEG-NH_2_	4-arm-polyethylene glycol-amine
8-arm-PEG-SG	8-arm-polyethylene glycol-succinimidyl glutarate
AKT	Protein kinase B
Alg-CH	Alginate-chitosan
ALP	Alkaline phosphatase
ATP	adenosine triphosphate
β-TCP	Beta-tricalcium phosphate
BV/TV	Bone volume/total volume
CAPA	Calcium alginate/poly acrylamide
CMC	Carboxymethyl chitosan
CPM	Carboxymethyl chitosan/polyaldehyde dextran-melatonin
DFDBA	Demineralized freeze-dried bone allograft
DPPH	2,2-diphenyl-1-picrylhydrazyl
EC	Evidence category
ECM	Extracellular matrix
EPS	Extracellular polymeric substance
F127DA	Pluronic F127 diacrylate
GCF	Gingival crevicular fluid
Gel-Alg	Gelatin-alginate
GelMA	Methacryloyl gelatin
GSH	Reduced glutathione
H&E	Hematoxylin and Eosin
HA	Hyaluronic acid
HAMA	Methacrylated hyaluronic acid
MHF	Methacrylated hyaluronic acid-pluronic F127 diacrylate
hPDLSC	Human periodontal ligament stem cell
IL	Interleukin
LPS	Lipopolysaccharide
M2-exos	M2 macrophage-derived exosomes
MBG	Mesoporous bioactive glass
MDA	Malondialdehyde
MFN2	Mitofusin 2
MINO	Minocycline
MMP	Matrix metalloproteinase
MPO	Myeloperoxidase
MT	Melatonin
mTOR	Mammalian target of rapamycin
MyD88	Myeloid differentiation factor 88
N/A	Not applicable
NF-κB	Nuclear factor-κB
Nrf2	Nuclear factor erythroid 2-related factor 2
NET	Neutrophil extracellular trap
OCN	Osteocalcin
OD	Oxidized dextran
OPG	Osteoprotegerin
OS	Oxidative stress
PAM	Poly acrylamide
PAP	Polylactide-b-aniline pentamer
PDLSC	Periodontal ligament stem cell
PEG	Polyethylene glycol
PI3K	Phosphoinositide 3-kinase
PTD	Polyvinyl alcohol/N^1^-(4-boronobenzyl)-N^3^-(4-boronophenyl)-N^1^,N^1^,N^3^,N^3^-tetramethylpropane-1,3-diaminium
PVA	Polyvinyl alcohol
RANKL	Receptor activator of nuclear factor kappa-B ligand
Refs	References
rhFGF-2	Recombinant human fibroblast growth factor type 2
ROS	Reactive oxygen species
RUNX2	Runt-related transcription factor 2
SA	Sodium alginate
SEM	Scanning electron microscopy
SIRT	Sirtuin
SOD	Superoxide dismutase
SRP	Scaling and root planning
TLR4	Toll-like receptor 4
TMEM110	Transmembrane protein 110
TNF-α	Tumor necrosis factor-α
TOM20	Translocase of the outer mitochondrial membrane 20
YAP	Yes-associated protein
